# Salvage hypofractionated accelerated versus standard radiotherapy for the treatment of biochemical recurrence after radical prostatectomy (SHARE): the protocol of a prospective, randomized, open-label, superiority, multi-institutional trial

**DOI:** 10.1186/s13063-021-05708-5

**Published:** 2021-10-21

**Authors:** Geumju Park, Yeon Joo Kim, Hanjong Ahn, Won Park, Ji sung Lee, Young Seok Kim

**Affiliations:** 1 Department of Radiation Oncology, Inje University Haeundae Paik Hospital, Busan, Republic of Korea; 2 Department of Radiation Oncology, Asan Medical Center, University of Ulsan, College of Medicine, 88, Olympic-ro 43-gil, Songpa-gu, Seoul, 05505 Republic of Korea; 3 Department of Urology, Asan Medical Center, University of Ulsan, College of Medicine, Seoul, Republic of Korea; 4 Department of Radiation Oncology, Samsung Medical Center, Sungkyunkwan University School of Medicine, Seoul, Republic of Korea; 5 Clinical Research Center, Asan Institute for Life Science, Asan Medical Center, University of Ulsan, College of Medicine, Seoul, Republic of Korea

**Keywords:** Hypofractionated radiotherapy, Prostate cancer, Salvage radiotherapy

## Abstract

**Background:**

While several phase III trials have investigated the role of hypofractionated radiotherapy in the definitive treatment of localized prostate cancer, prospective data reporting the outcomes of hypofractionated radiotherapy in the postoperative treatment setting are sparse. Therefore, this study is designed to assess the efficacy and treatment-related toxicity of hypofractionated salvage radiotherapy for the treatment of biochemical recurrence in men who underwent radical prostatectomy. The primary objective of this trial is to investigate whether hypofractionated radiotherapy improves biochemical control compared with conventionally fractionated radiotherapy. In addition, treatment-related toxicity, quality of life, and survival will be evaluated as secondary endpoints.

**Methods:**

In this prospective, randomized, multi-institutional trial (the SHARE study), patients with intermediate- or high-risk prostate cancer will be randomized to receive either hypofractionated radiotherapy (65 Gy in 2.5-Gy fractions) or conventionally fractionated radiotherapy (66 Gy in 2-Gy fractions). Prostate bed irradiation or elective pelvic nodal irradiation including the prostate bed will be performed using intensity-modulated radiotherapy and daily image guidance. Treatment efficacy will be assessed using the serum tumor marker prostate-specific antigen, and toxicity will be evaluated through both physician- and patient-reported outcomes. Quality of life will also be investigated.

**Discussion:**

This study is designed to demonstrate whether hypofractionated radiotherapy is beneficial in terms of biochemical control and toxicity compared with standard salvage radiotherapy. If hypofractionated radiotherapy is shown to be superior to conventionally fractionated radiotherapy, it will mean that improved biochemical control can be achieved, accompanied by greater patient convenience and more efficient use of medical resources.

**Trial registration:**

ClinicalTrials.gov NCT03920033. Registered on 18 April 2019

## Background

After radical prostatectomy for the treatment of localized prostate cancer, approximately one-third of men will subsequently experience biochemical recurrence within a decade [1]. The risk of a rise in prostate-specific antigen (PSA) level was > 60% in patients with adverse high-risk features, such as Gleason score ≥ 8, serum PSA > 20 ng/mL, or stage of T3 or T4 [2]. Salvage radiotherapy (RT) is a potentially curative treatment option for biochemical recurrence following radical prostatectomy [3, 4].

Dose-escalated RT (76–80 Gy, 1.8–2.0 Gy per fraction) became the standard treatment for intact localized prostate cancer after several randomized trials demonstrated improved biochemical progression-free survival (bPFS) with this approach [5–7]. However, the potential benefit derived from dose-escalation in the postoperative setting remains to be clarified. The ASTRO/AUA guidelines recommend a minimum dose of 64–65 Gy with conventional fractionation in the postoperative setting, as the presumption is that the tumor burden is microscopic after prostatectomy, and therefore, a lower dose will be required in the postoperative setting versus the definitive setting [8]. However, several retrospective analyses have suggested that dose escalation during salvage RT may lead to improved biochemical control [9–13]. In a systematic review of 10,034 patients from 71 studies, the author noted that the dose response of salvage RT was similar to that of definitive RT of localized disease, and each increase of 1 Gy led to a 2% improvement in relapse-free survival [9]. Pisansky et al. evaluated 1108 patients who underwent salvage RT at 10 academic centers, demonstrating that doses > 66 Gy achieved superior biochemical control [13]. To date, the only phase III randomized clinical trial testing the dose escalation for salvage RT has been conducted by the Swiss Group for Clinical Cancer Research (SAKK). The SAKK 09/10 study compared dose-escalated RT (70 Gy in 35 fractions) versus standard-dose RT (64 Gy in 32 fractions) in the salvage setting. The initial results showed that dose escalation up to 70 Gy was associated with low rates of acute toxicity and had no impact on the recovery of early urinary continence or the prevalence of de novo incontinence [14, 15]. Freedom from biochemical failure, a primary endpoint of this trial, has not yet been reported.

For prostate cancer, pre-clinical and clinical data have shown that an *α*/*β* ratio of a tumor is low [16–18]. The *α*/*β* ratio of prostate cancer is 1.4–1.9 Gy, lower than the > 3 Gy reported for the surrounding normal tissues. This suggests that RT using hypofractionation (larger fraction size and fewer fractions) would increase the probability of tumor control without increasing treatment-related toxicities. In addition, recent advances in RT techniques, such as intensity-modulated RT (IMRT) and image-guided RT, have increased the potential for normal tissue sparing and improved toxicity [19, 20]. In the definitive setting, randomized trials of patients with prostate cancer receiving hypofractionated RT (HRT) versus conventionally fractionated RT (CRT) showed non-inferior or superior cancer control with HRT [21–25]. However, few randomized studies have evaluated the impact of HRT in patients with biochemical recurrence after prostatectomy. Several retrospective and phase I/II studies reported that HRT was tolerable in terms of toxicity and demonstrated encouraging results for cancer control in the postoperative setting [26–36]. The results of HRT using modern techniques, such as IMRT, volumetric-modulated arch therapy, or helical tomotherapy, in the postoperative setting are described in Tables 1 and 2. To date, despite clinical data suggesting a dose-response relationship in the salvage RT and radiobiological aspect of a low *α*/*β* ratio of prostate cancer, no randomized controlled trials have compared HRT versus CRT in the salvage setting.

**Table 1:** Literature review of hypofractionated radiotherapy schemes for the treatment of prostate cancer in the postoperative setting

Study (ref.)	Design	Setting (patient number)	Total dose, Gy	Dose per fraction, Gy	EQD2 (prostate cancer^a^)	BED (early toxicity^a^)	BED (late toxicity^a^)
Tandberg [30]	Retrospective	Salv. (138)/Adj. (29)	65^b^	2.5	74.29	81.25	119.17
Kruser [33]	Retrospective	Salv. (108)	65	2.5	74.29	81.25	119.17
Lewis [31]	Retrospective	Salv. (43)/Adj. (13)	65^b^	2.5	71.43	78.13	114.58
Cuccia [32]	Retrospective	Salv. (38)/Adj. (37)	63.8	2.2	67.45	77.84	110.59
Barra [34]	Retrospective	Salv. (32)/Adj. (32)	62.5	2.5	71.43	78.13	114.58
Alongi [35]	Retrospective	Salv. (9)/Adj. (30)	70^b^	2.5	80	87.5	128.33
Saldi [27]	Phase I/II	Salv. (72)/Adj. (40)	72–74.25^c^	2.25	77.14–79.55	88.2–90.96	126–129.94
Katayama [26]	Phase I/II	Salv. (28)/Adj. (11)	54	3	69.43	70.2	108
Gladwish [29]	Phase I/II	Salv. (26)/Adj. (4)	51	3	65.57	66.3	102
Macchia [28]	Phase I/II	Salv. (18)/Adj. (106)	62.5	2.5	71.43	78.13	114.58
This study	Phase III	Salv. (288)	65	2.5	74.29	81.25	119.17

*EQD2* equivalent dose in 2-Gy fractions, *BED* biologically equivalent dose, *Salv.* salvage, *Adj.* adjuvant

^a^*α*/*β* ratio for prostate cancer is 1.5, 10 for early-responding normal tissue, and 3 for late-responding normal tissue

^b^Median

^c^Salvage

**Table 2:** Literature review of hypofractionated radiotherapy outcomes for the treatment of prostate cancer in the postoperative setting

Study (ref.)	Median follow-up, months	bPFS, %	Toxicity scoring system	Acute toxicity, %		Late toxicity, %	
				GU	GI	GU	GI
Tandberg [30]	38.6	78.4^a^ (4 years)	CTCAE v4.0^b^/RTOG^c^	≥ G2: 22; G3: 1^d^	G2: 5; ≥ G3: 0	G2: 39; ≥ G3: 11	G2:10; G3: 1^d^
Kruser [33]	32.4	67 (4 years)	RTOG	G2: 6.4; G3: 1^d^	G2: 14; ≥ G3: 0	G2: 15; ≥ G3: 0	G2: 4; ≥ G3: 0
Lewis [31]	48	75 (4 years)	CTCAE v4.0^b^/RTOG^c^	G2: 4; ≥ G3: 0	G2: 4; ≥ G3: 0	G2: 39; G3: 28	≥ G3: 2^d^
Cuccia [32]	30	73 (3 years)	CTCAE v4.0	G2: 4; ≥ G3: 0	G2: 18; ≥ G3: 0	G2: 2.6; G3: 2.6	G2: 6.6; ≥ G3: 0
Barra [34]	15.5	Not reported	CTCAE v4.0^b^/RTOG^c^	≥ G2: 0	≥ G2: 0	G2: 3.3; G3: 3.3	≥ G2: 0
Alongi [35]	22.8	Not reported	RTOG	G2: 10; ≥ G3: 0	G2: 20; ≥ G3: 0	G2: 8; G3: 3	≥ G2: 0
Saldi [27]	27	Not reported	CTCAE v4.0	G2: 8.9; ≥ G3: 0	G2: 9.8; ≥ G3: 0	Not reported	Not reported
Katayama [26]	Not reported	Not reported	CTCAE v4.0	≥ G2: 0	G2: 17.9; ≥ G3: 0	Not reported	Not reported
Gladwish [29]	24	Not reported	CTCAE v3.0^b^/RTOG^c^	G2: 3; G3: 3	≥ G2: 0	≥ G2: 1^d^	≥ G2: 2^d^
Macchia [28]	30	86.5 (5 years)	RTOG	G2: 17.7; G4: 1^d^	G2: 24.2; ≥ G3: 0	≥ G2: 7.3	≥ G2: 1.1

*bPFS* biochemical progression-free survival, *GU* Genitourinary, *GI* gastrointestinal

^a^Patients with a 12-month minimum follow-up were analyzed for bPFS

^b^Acute

^c^Late

^d^Number of patients

The protocol of the Salvage hypofractionated accelerated versus standard radiotherapy for biochemical recurrence after radical prostatectomy (SHARE) study is outlined here. This prospective, randomized, multi-institutional trial is designed to assess whether HRT improves biochemical control versus CRT without increasing treatment toxicity in prostate cancer patients with biochemical recurrence after radical prostatectomy. This study uses two RT dose schemes, HRT (65 Gy in 2.5-Gy fractions) versus CRT (66 Gy in 2-Gy fractions).

## Methods/design

### Recruitment and study design

**Fig. 1:**
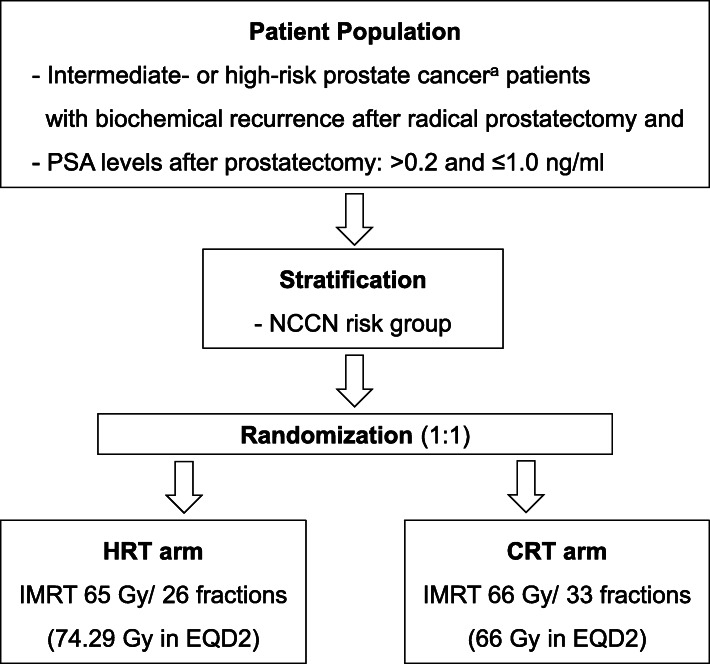
Flowchart of study treatments. ^a^Risk groups defined according to the National Comprehensive Cancer Network (NCCN) guidelines. PSA, prostate-specific antigen; HRT, hypofractionated radiotherapy; CRT, conventionally fractionated radiotherapy; IMRT, intensity-modulated radiotherapy; EQD2, equivalent dose in 2-Gy fractions

The SHARE study is a prospective, randomized, multi-institutional trial. The protocol was approved by the institutional review boards of two participating tertiary hospitals, the Asan Medical Center and Samsung Medical Center, where it is currently ongoing. A computer-generated randomization schedule assigns the patients to either the HRT (65 Gy in 2.5-Gy fractions) or CRT (66 Gy in 2-Gy fractions) arm (Fig. 1). Random assignment is performed with a 1:1 allocation using blocked randomization, with a random block size of 4. Patients will be stratified by intermediate or high-risk according to the National Comprehensive Cancer Network (NCCN) risk classification [37] using a computer-generated random allocation sequence to ensure concealment. The clinical research nurse keeps the original random allocation sequences using the Excel file locked with a password. The process of allocation concealment from primary researchers and participants is maintained until the time of assignment. Neither the investigators nor the participants are masked to the allocated arm because blinding is not possible. For adequate participant recruitment, the investigators fully explain to potential participants the purpose, design, potential risks, and benefits of this study, as well as general ethical issues. A monthly newsletter regarding participant enrollment will be made and sent to the principal investigator and clinical research coordinators of each institution. All patients must provide written informed consent.

As summarized in Table 3, the equivalent dose in 2-Gy fractions (EQD2) for the HRT regimen is 74.29 Gy, assuming an *α*/*β* ratio of 1.5 Gy for prostate cancer [38]. The HRT arm delivers a higher biologically effective dose (BED) than the CRT arm (173.33 Gy vs. 154 Gy). Assuming an *α*/*β* ratio of 10 Gy for acute-responding normal tissue, the BED is 81.25 Gy for the HRT arm versus 67.71 Gy for the CRT arm. Using an *α*/*β* ratio of 3 Gy for late-responding normal tissue, the HRT arm resulted in a BED of 119.17 Gy, which is slightly higher than the 110 Gy for the CRT arm.

**Table 3:** Equivalent dose in 2-Gy fractions (EQD2) and biologically effective dose (BED) calculations

	Conventionally fractionated (66 Gy in 33 fractions)	Hypofractionated (65 Gy in 26 fractions)
EQD2^a^ (Gy)		
Prostate cancer	66	74.29
Early-responding normal tissue	66	67.71
Late-responding normal tissue	66	71.5
BED^a^ (Gy)		
Prostate cancer	154	173.33
Early-responding normal tissue	79.2	81.25
Late-responding normal tissue	110	119.17

^a^*α*/*β* ratio for prostate cancer is 1.5, 10 for early-responding normal tissue, and 3 for late-responding normal tissue

### Inclusion criteria

Eligible patients are required to have a diagnosis of histologically confirmed prostate adenocarcinoma after radical prostatectomy and must fulfill the following criteria: aged ≥ 19 years, an Eastern Cooperative Oncology Group performance status 0–1, intermediate or high risk according to the NCCN guidelines [37], and total serum PSA level after prostatectomy > 0.2 and ≤ 1.0 ng/ml. Patients should also have adequate bone marrow (absolute neutrophil count ≥ 1500 cells/mm^3^; hemoglobin concentration ≥ 8.0 g/dl; platelet count ≥ 50,000 cells/mm^3^), liver (total bilirubin < 1.5 times the maximum normal value; alanine aminotransferase or aspartate aminotransferase < 2.5 times the maximum normal value), and renal (creatinine < 2.0 ng/dl) function, as assessed by tests performed within the 6-month period prior to enrollment.

### Exclusion criteria

Patients are not eligible for inclusion if they have gross nodules detected in the prostate bed; clinical, radiographic, or pathologic evidence of nodal disease; presence of distant metastasis; prior neoadjuvant chemotherapy, cryosurgery, or brachytherapy of the prostate; prior pelvic RT or chemotherapy for any other disease; and any invasive malignancy diagnosed within 5 years of entry or if they have a severe active comorbidity.

### Dropout criteria

Patients shall be free to withdraw their consent once given but shall not suffer any disadvantage in terms of medical practice for the withdrawal. Any participant with medical problems or unexpected serious complications occurring during treatment or follow-up period that means the study protocol cannot be completed will be analyzed on an intention-to-treat basis.

### Treatment implementation

Androgen deprivation therapy (ADT) is prescribed at the discretion of the investigator and/or physicians; administration of luteinizing hormone-releasing hormone agonists and/or anti-androgens is permitted in both treatment arms.

For RT planning, a computed tomography (CT) scan of the pelvis with a slice thickness of 2.5 mm will be obtained in all patients. Participants will be scanned in a supine position immobilized with an ankle pillow and instructed to make the bladder volume constant (empty or retain a certain amount of urine); a rectal balloon will be inserted prior to CT simulation and each treatment. There will be no routine bowel preparation. Clinical target volume (CTV) includes the prostate bed according to the Radiation Therapy Oncology Group (RTOG) contouring consensus guidelines [39]. At the discretion of the treating clinician, the pelvic lymph nodes can be included in the target volume. The planning target volume (PTV) is defined as an expansion of the CTV by 7 mm in all dimensions, other than posteriorly which will be 3–5 mm. The critical organs that are contoured include the rectum, bladder, femoral heads, penile bulb, and small bowel. The entire rectum is contoured from the anus to the rectosigmoid flexure. All patients will be treated with IMRT, and ≥ 95% of the PTV must receive the prescription dose. The use of daily image-guided techniques is strongly recommended but not mandatory.

Patients assigned to the HRT arm will receive 65 Gy of radiation in 26 fractions of 2.5 Gy (five fractions per week); for the CRT arm, 66 Gy of radiation will be administered in 33 fractions of 2 Gy (five fractions per week). The two arms differ only in the fractionation scheme. In cases of elective irradiation of the pelvic lymph nodes, 45–50 Gy will be administered using a simultaneously integrated boost technique in both arms.

### Data collection and follow-up period

**Fig. 2:**
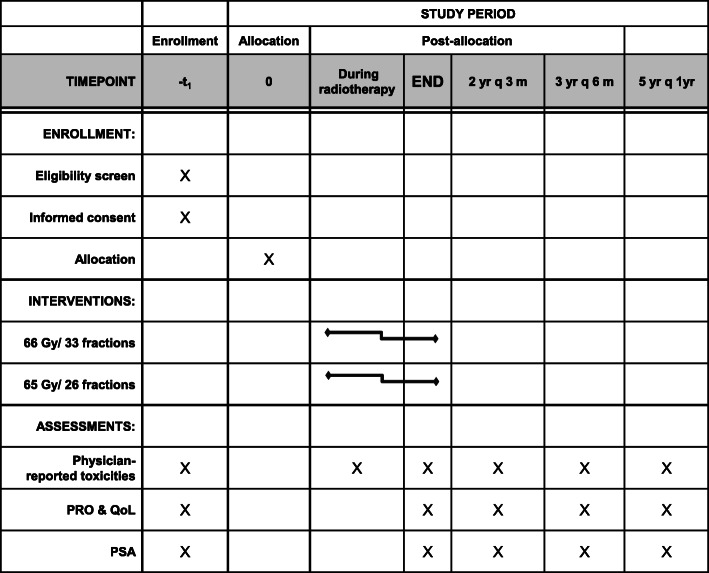
Intervention and assessment schedule for the trial according to the Recommendations for Interventional Trials (SPIRIT). PRO, patient-reported outcomes; QoL, quality of life; PSA, prostate-specific antigen

Patient data will be collected according to the schedule shown in Fig. 2, i.e., prior to initiating RT, weekly during RT, every 3 months for the first 2 years after RT, every 6 months for years 3–5, and then annually thereafter. The following patient data will be recorded in case report forms: urologic/gastrointestinal (GI) toxicities using the Common Toxicity Criteria for Adverse Events (CTCAE v5.0), patient-reported outcomes, QoL, and PSA levels. A pop-up sticker note will be generated automatically on the electronic medical record system to improve adherence to intervention protocols and follow-up laboratory tests. These outcome data will be aggregated as median and interquartile range. If tumor recurrence or metastases are suspected, pelvic magnetic resonance imaging, CT, and/or bone scans can be performed at the discretion of the investigator.

To ensure patient data protection, separate identification codes will be given to all patients. Data will password-protected and only accessible by investigators. All data will be deleted 3 years after the end of the study.

### Assessment of primary and secondary endpoints

The primary endpoint is to investigate whether HRT improves 5-year bPFS versus CRT in patients with biochemical recurrence after radical prostatectomy. Biochemical progression-free survival is counted from the date of the salvage radiotherapy to biochemical progression, clinical progression, or death of any cause. Biochemical progression after RT is defined as detectable PSA after salvage RT. Secondary endpoints are to compare acute and late toxicities based on CTCAE v5.0 and QoL and to determine 5-year distant metastasis-free survival (DMFS) and cancer-specific survival (CSS).

### Sample size considerations and statistical analysis

The trial is powered to assess the superiority of HRT when compared with CRT. Power calculations are based on an absolute increase in bPFS of 15% (from 50 to 65%) at 5 years following HRT compared with CRT according to the dose-response relationship described by King [9]. By postulating a minimum 5-year follow-up period and allowing 5% for loss, at least 144 patients per treatment arm (288 patients in total) are required, which achieves 80% power, with a two-sided *α* of 0.05. Subgroup analyses will be performed based on the risk group (intermediate vs. high-risk group), RT field size (prostate bed only vs. elective pelvic nodal irradiation), and use of ADT.

Statistical analysis will be conducted according to the intention-to-treat approach. Survival data, PSA value, toxicity, and QoL information will be collected even though participants discontinue the planned treatment. Multiple imputation method will be used for handling missing data. The chi-square test and Student’s *t* test will be used for categorical and continuous variables of patient characteristics, respectively. Time to biochemical progression will be calculated from the end of RT. The Kaplan–Meier method will be applied to estimate bPFS curves, and the log-rank test will be used to compare the curves between the two study arms. The Kaplan-Meier method and log-rank test will be used to investigate DMFS and CSS. Acute toxicity outcomes and QoL will be estimated using the chi-square test, and late toxicity outcomes will be determined using the Kaplan-Meier method.

## Discussion

By increasing the radiobiological dose delivered to the prostate, HRT has the potential to shorten the duration of treatment, thereby improving patient convenience and reducing healthcare costs. In the past two decades, HRT in the intact prostate cancer setting has been evaluated in randomized controlled trials, demonstrating this approach as a safe and effective alternative to CRT. However, the efficacy and toxicity of HRT in the postoperative treatment setting remain to be validated. Several retrospective or phase I/II studies of HRT in the postoperative setting have used heterogeneous dose schemes and toxicity scoring systems, making it difficult to interpret the results. No data from randomized studies of HRT in the management of patients with biochemical recurrence after radical prostatectomy are currently available.

Biochemical control after HRT using modern RT techniques in the postoperative setting has been seen to be favorable in several studies. The studies by Tandberg et al., Kruser et al., and Lewis et al. used a HRT scheme of 65 Gy in 2.5-Gy fractions (EQD2 = 74.29 Gy; *α*/*β* ratio = 1.5 Gy), demonstrating a 4-year bPFS of 78.4, 67, and 75%, respectively [30, 31, 33]. In particular, Tandberg et al. retrospectively compared oncologic outcomes between HRT (*n* = 167) and CRT (*n* = 294) [30]. With a median follow-up of 52 months, 4-year bPFS was 78.4% after HRT and 64.8% after CRT (*P* = 0.0038). On multivariate analysis, there was a trend for decreased risk of biochemical progression in the HRT group compared with the CRT group (*P* = 0.059). Cuccia et al. reported a 3-year bPFS of 73% in 75 patients treated with salvage or adjuvant aims, but the prescribed dose (63.8 Gy in 2.2-Gy fractions; EQD2, 67.45 Gy) was relatively low compared with those used in other HRT studies [32]. The phase I/II study by Macchia et al. used RT with the 62.5 Gy in 2.5-Gy fractions scheme (EQD2 = 71.43 Gy) and reported a 5-year bPFS of 86.5% [28]. However, the study included a relatively small number of patients receiving RT with a salvage aim (18 of 124 patients). Therefore, to date, significant heterogeneity in the dose scheme and treatment aims (both salvage and adjuvant) means that it is difficult to draw conclusions regarding the oncologic treatment outcomes of salvage HRT.

Genitourinary (GU) and GI toxicities are considered to be key obstacles to the administration of HRT for prostate cancer in the postoperative setting. Although different toxicity scoring systems used in previous studies have made it difficult to assess the safety of HRT in the postoperative setting, most have demonstrated a tolerable toxicity profile [26–28, 30, 31, 33–36]. Kruser et al. (65 Gy in 2.5-Gy fractions) reported low rates of toxicity (one acute grade 3 GU toxicity, no acute grade 3 GI toxicities, and no late grade 3 toxicities) in 108 salvage RT patients [33]. Tandberg et al. evaluated HRT (65 Gy in 2.5-Gy fractions) in 167 patients and CRT (66 Gy in 1.8–2.0-Gy fractions) in 294 patients to determine any differences in toxicity between the two treatment groups [30]. The results showed that acute grade ≥ 2 GU toxicity was more common after HRT (22 vs. 8% in the CRT group, *P* = 0.0001), but HRT was not associated with late grade ≥ 2 GU toxicity on multivariate analysis. In the study by Barra et al., late grade 3 GU toxicity was recorded in 3.3% of 64 patients who received RT with a dose scheme of 62.5 Gy in 2.5-Gy fractions [34]. Alongi et al. reported no toxicities ≥ grade 3 in 39 patients who received RT with a median dose of 70 Gy in 2.5-Gy fractions [35]. A single acute grade 4 GU toxicity event was reported by Macchia et al. (62.5 Gy in 2.5-Gy fractions) [28]. In the Lewis et al. study, toxicity events > grade 3 were unusual in patients with a median dose of 65 Gy in 2.5-Gy fractions, although a relatively high rate of late GU toxicity was seen, noting a 4-year actuarial rate of late grade 3 GU toxicity of 28%, which was exclusively gross hematuria [31]. With a median follow-up of 48 months, most cases of grade 3 GU toxicity resolved, with only 7% persisting at the last follow-up visit. Analyses of bladder dose volume and PTV volume revealed no significant differences between patients with and without grade 3 GU toxicity. The authors suggested that potential explanations for the high rate of late GU toxicity could include the longer follow-up times and differences in treatment imaging and radiation delivery versus other studies, although the precise reason was not clear. Acute and late toxicities associated with HRT in the postoperative setting were relatively acceptable in most studies.

As phase III trials have demonstrated the benefits of ADT in the definitive RT setting, the addition of ADT to salvage RT may be beneficial in some patients. However, the optimal timing, duration, and type of hormone treatment remain to be determined. Two phase III trials advocated the use of ADT for patients who received salvage RT. The RTOG-9601 trial, a placebo-controlled phase III study, demonstrated that the addition of 24 months’ ADT with daily bicalutamide to salvage RT resulted in significantly higher rates of overall survival (76.3 vs. 71.3% in patients receiving placebo; *P* = 0.04), and a lower incidence of metastatic prostate cancer (14.5 vs. 23%, respectively; *P* = 0.005), and death from prostate cancer (5.8 vs. 13.4%, respectively; *P* < 0.001) at 12 years [40]. Another randomized trial, GETUG-AFU 16, has also shown the superiority of adding 6 months of goserelin to salvage RT [41, 42]. After a median follow-up of 112 months, the 10-year progression-free survival was significantly better in the ADT arm versus RT alone (64 vs. 49%, respectively; *P* < 0.0001). The RADICALS trial randomly assigned patients to adjuvant RT or salvage RT, with or without ADT, and the NRG Oncology/RTOG 0534 SPPORT trial randomly assigned patients to salvage RT to the prostate bed, salvage RT to the prostate bed with ADT, or salvage RT to the prostate bed and the pelvic lymph nodes with ADT. The results of these trials could provide further evidences concerning the administration of postoperative ADT. To date, it is uncertain whether ADT in combination with salvage HRT improves biochemical control. In the trial outlined here, the authors will conduct subgroup analyses according to the use of ADT and examine the benefit derived from combined therapy. We anticipate that HRT will demonstrate favorable biochemical control and acceptable toxicity in prostate cancer patients with biochemical recurrence after radical prostatectomy. If HRT is seen to be superior to CRT, it will indicate the benefit of escalating dose delivery to the prostate bed, which will also improve patients’ convenience and reduce healthcare costs.

## Trial status

The current protocol is version 1.6 as of 4 February 2021. Patient recruitment began in August 2019 and is expected to be completed by March 2022.

## Data Availability

The datasets used during the current study are available from the corresponding author on reasonable request.

## Abbreviations

PSA: Prostate-specific antigen
RT: Radiotherapy
bPFS: Biochemical progression-free survival
SAKK: Swiss Group for Clinical Cancer Research
IMRT: Intensity-modulated radiotherapy
HRT: Hypofractionated radiotherapy
CRT: Conventionally fractionated radiotherapy
QoL: Quality of life
NCCN: National Comprehensive Cancer Network
EQ D2: Equivalent dose in 2-Gy fractions
BED: Biologically effective dose
ADT: Androgen deprivation therapy
CT: Computed tomography
CTV: Clinical target volume
PTV: Planning target volume
GI: Gastrointestinal
CTCAE: Common Toxicity Criteria for Adverse Events
DMFS: Distant metastasis-free survival
CSS: Cancer-specific survival
GU: Genitourinary
